# Multisensor Characterization of Process Behavior During Underwater Laser Cutting of 100 mm Thick STS 304 Stainless Steel

**DOI:** 10.3390/s26206383

**Published:** 2026-10-09

**Authors:** Seong Jun Mun, Jung Soo Choi, Young Whan Park, Tae Wan Kim

**Affiliations:** 1 Department of Intelligent Robot Engineering, Pukyong National University, Busan 48513, Republic of Korea; munsj@pukyong.ac.kr; 2 Laser Industrial Technology Research Group, Korea Institute of Machinery & Materials, Busan 46744, Republic of Korea; cjs1989@kimm.re.kr; 3 Department of Mechanical Engineering, Pukyong National University, Busan 48513, Republic of Korea

**Keywords:** underwater laser cutting, multisensor monitoring, nuclear decommissioning

## Abstract

Underwater laser cutting is a promising remote segmentation method for highly activated reactor internals because it enables non-contact cutting while maintaining water shielding. Although previous studies have demonstrated the feasibility of thick-section underwater laser cutting, process monitoring remains challenging because bubbles, optical scattering, local gas-cavity behavior, water cooling, and complex melt ejection can alter the relationship between the cutting state and externally measured signals. Therefore, the monitoring characteristics of different sensing modalities during underwater thick-section cutting need to be clarified. This study synchronized a camera, a broadband photodiode, and a triaxial accelerometer during underwater laser cutting of 100 mm thick STS 304 stainless steel. Camera observations were used as the reference for identifying process states. During partial penetration, a broad lateral plume developed, followed by transient lower-lateral ejection and pronounced downward discharge at full penetration. The photodiode mean decreased by 88.8% and 93.0% from partial to full penetration in Experiments 1 and 3, respectively, and remained low afterward. Accelerometer responses around full penetration were less consistent, whereas in Experiment 2, late-stage abnormal behavior was accompanied by a 128.8% increase in the mean PSD in the 75–245 Hz band. These results indicate distinct monitoring characteristics of the three sensing modalities.

## 1. Introduction

Nuclear facility decommissioning requires not only the safe removal of activated structures and the management of radioactive waste, but also the minimization of radiation exposure to workers and environmental impacts. In particular, remote-operation technologies are required to safely cut and dismantle large structures while reducing direct worker access in high-radiation environments. The International Atomic Energy Agency (IAEA) has emphasized the increasing global demand for nuclear decommissioning, together with the importance of remote operation, technological advancement, and efficient segmentation and waste-management strategies [1].

In light-water reactor (LWR) decommissioning, reactor internals are particularly challenging to dismantle because they are highly activated, have large section thicknesses and complex geometries, and are subject to restricted accessibility. During segmentation of highly activated internal structures, water shielding is often maintained to reduce worker radiation exposure; consequently, the cutting process is performed underwater. Therefore, a practically applicable segmentation technology must provide sufficient cutting capability for thick metallic components while also being suitable for remote operation and stable underwater processing.

Laser cutting is well suited to this purpose because a high-power beam can be delivered remotely through optical fiber, the process is non-contact, and the narrow kerf can reduce material loss. Underwater operation, however, requires the formation of a local dry zone for laser-beam transmission and sufficient assist-gas momentum to expel molten material against the resistance of the surrounding water. Matsumoto et al. proposed an early underwater cutting method using a local-dry-zone creating nozzle [2]. Hilton and Khan demonstrated the applicability of a fiber-deliverable 1 μm laser source to underwater cutting [3]. Leschke et al. investigated the robustness of underwater laser cutting of structural steel using a Yb:YAG disk laser [4]. Subsequent studies extended underwater laser cutting to thicker stainless-steel sections. Shin et al. demonstrated underwater cutting of 50–60 mm thick stainless steel using a 6 kW fiber laser [5]. In a subsequent study, Shin et al. reported underwater laser cutting of stainless steel up to 100 mm in thickness using a higher-power system [6]. They later investigated an initial oblique-cutting strategy to improve the underwater cutting of thick steel plates [7]. Kim et al. examined the effects of major process conditions on the underwater cutting characteristics of 50 mm thick stainless steel [8]. Song et al. evaluated the cutting performance of 100 mm thick AISI 304 under a simulated water depth of 10 m while considering different cutting speeds [9]. These studies established the feasibility of thick-section underwater laser cutting, but their evaluations focused mainly on achievable cutting thickness and speed, process conditions, kerf geometry, and post-process cut quality.

Externally measured signals during laser cutting are closely related to material-removal behavior within the kerf. Hirano and Fabbro experimentally investigated the melt-layer dynamics during laser cutting of steel and showed that melt-layer instability and discontinuous lateral flow are associated with striation formation [10]. Pocorni et al. investigated the fluid dynamics and cut-front morphology during fiber-laser cutting of stainless steel and related melt-film flow and cut-front behavior to dross formation and cut-surface characteristics [11]. These studies suggest that changes in cut-front geometry, molten-material ejection, and gas–melt interaction can be reflected in changes in optical emission and mechanically transmitted responses. Therefore, in underwater thick-section cutting, externally measurable signals are relevant not only for identifying whether material removal is occurring, but also for understanding how the process progresses toward full penetration and changes thereafter.

Sensor-based monitoring has been investigated mainly for conventional in-air laser cutting. Garcia et al. analyzed photodiode responses under different laser-cutting conditions and showed that optical signals can provide information on process variations [12]. Levichev et al. performed photodiode-based real-time monitoring during fiber-laser cutting of thick plates and reported signal changes associated with penetration and process conditions [13]. Kek and Grum investigated acoustic emission during laser cutting and related acoustic-emission characteristics to cutting events and cut quality [14]. However, these findings cannot be directly transferred to underwater thick-section cutting because bubbles, local gas-cavity behavior, water cooling, and complex molten-material ejection modify the physical environment in which material removal and signal generation occur. Consequently, the relationships between cutting-process progression and externally measured signals established for conventional in-air cutting may not remain unchanged under underwater conditions, requiring direct characterization under the actual underwater cutting environment.

Thus, previous studies have demonstrated the applicability of thick-section underwater laser cutting and the potential of sensor-based process monitoring; however, the relationship between cutting-process progression and externally measured sensor responses under underwater conditions has not yet been sufficiently characterized. In this study, underwater laser-cutting experiments were conducted on 100 mm thick STS 304 stainless steel, and signals from a camera, a broadband photodiode, and a triaxial accelerometer were synchronized and analyzed. Major process states were identified using camera observations as a reference, and the corresponding photodiode and accelerometer responses were compared on the same time basis. Through this approach, the study aimed to characterize how externally measured sensor responses changed with penetration progression and late-stage abnormal behavior and to clarify the monitoring characteristics of the three sensing modalities under underwater thick-section cutting conditions.

## 2. Materials and Methods

### 2.1. Experimental Setup and Multisensor Data Acquisition

**Figure 1:**
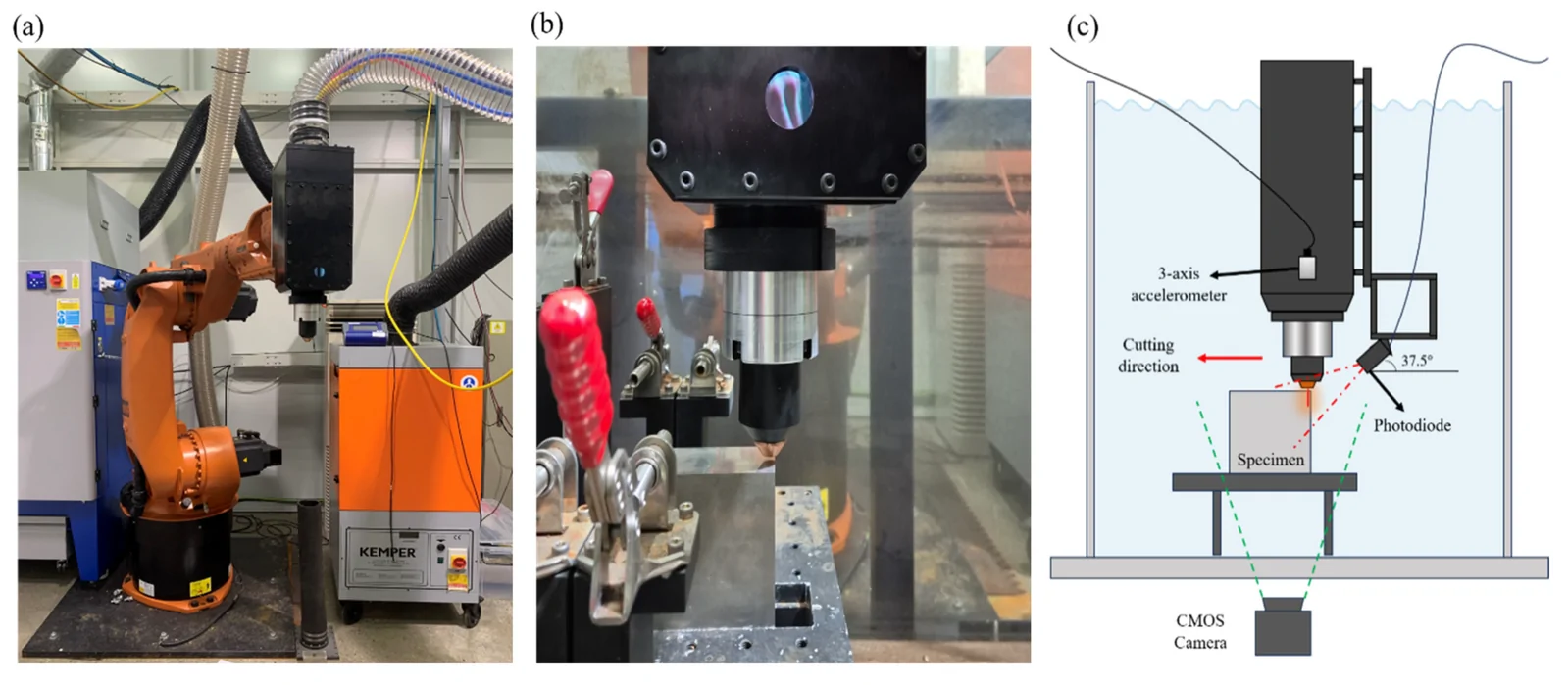
Underwater laser-cutting experimental system: (**a**) laser cutting head and robotic system; (**b**) close-up arrangement of the cutting head and specimen; (**c**) schematic of the camera, photodiode, and accelerometer locations and observation geometry.

Underwater laser cutting was performed on 100 mm thick STS 304 stainless steel using a 30 kW-class fiber-laser system (Raycus, Wuhan, China) operated at 22 kW. The specimen was positioned at an approximately 1 m water depth. Nitrogen was supplied as the assist gas at 5 bar to maintain a local dry zone near the laser–material interaction region and to promote molten-material ejection. The nozzle tip was positioned approximately 5 mm above the upper surface of the specimen. The cutting speed was set to 12 mm min^−1^ over the initial 30 mm of the cutting path to facilitate reliable cutting initiation and initial kerf formation, and was subsequently increased to 30 mm min^−1^ for the remaining cutting distance. The focal position was set to −40 mm relative to the upper surface of the specimen. The same cutting conditions and two-stage cutting-speed profile were applied to all analyzed experiments. The overall experimental arrangement and sensor installation are shown in Figure 1, and the principal cutting and underwater process conditions are summarized in Table 1.

Three synchronized cutting experiments performed under these nominal process conditions were analyzed. Experiments 1 and 3 provided valid camera, photodiode, and accelerometer records and were used for synchronized multisensor analysis of the cutting process. Experiment 2 provided synchronized camera and accelerometer records; the photodiode record was unavailable and was therefore excluded from the photodiode analysis. Unlike Experiments 1 and 3, Experiment 2 exhibited a distinct late-stage change in plume behavior after full penetration. Therefore, Experiment 2 was analyzed separately to characterize the corresponding camera and accelerometer responses during this late-stage abnormal behavior. Given the limited number of experiments, the present analysis is exploratory and is intended to characterize the observed sensor-response tendencies rather than to establish repeatability or predictive capability.

**Figure 2:**
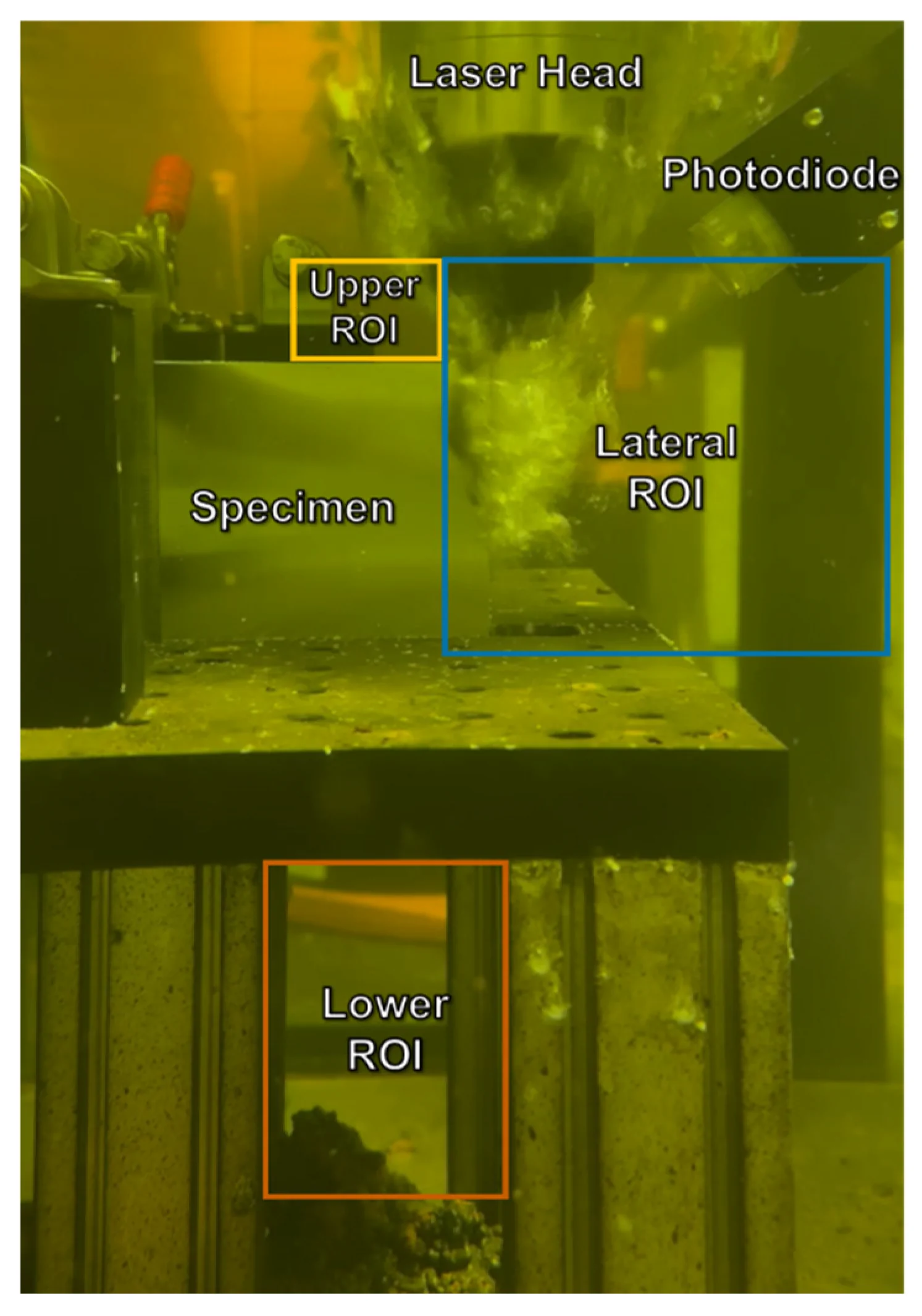
Camera observation regions and ROIs used for quantitative image analysis.

A CMOS camera FL3-U3-32S2C-CS (FLIR Integrated Imaging Solutions Inc., Richmond, Canada) equipped with a M0814-MP2 lens (CBC Co., Tokyo, Japan) with an 8 mm focal length was installed outside the water tank and recorded the cutting region at a resolution of 1920 × 1080 pixels and 30 fps. Automatic exposure and automatic gain were enabled during acquisition, whereas automatic white balance was disabled. To distinguish the spatial evolution of the luminous plume during cutting, the field of view was divided into upper, lateral, and lower regions of interest (ROIs). The upper ROI covered the nozzle-side emission region, the lateral ROI covered the dominant side-emission region, and the lower ROI covered the area in which downward ejection below the specimen could be observed. The three ROIs are shown in Figure 2.

A broadband photodiode S12698-02 (Hamamatsu Photonics K.K., Hamamatsu, Japan) with a spectral response range of 190–1000 nm was positioned above and to the right of the cutting region, with its viewing direction inclined approximately 37.5° downward. The photodiode was installed in a watertight enclosure and directly viewed the cutting region through an approximately 120 mm underwater optical path. No additional optical filter was used. The photodiode output was amplified using an external amplifier and acquired at 200 Hz. A triaxial accelerometer with a nominal sensitivity of 100 mV/g was mounted directly on the cutting head and sampled at 5000 Hz. The vertical Z-axis signal was used for vibration analysis.

### 2.2. Time Synchronization and Definition of Cutting States

Because the camera, photodiode, and accelerometer were recorded by different acquisition systems and at different sampling rates, their time axes were aligned using laser-emission onset as a common synchronization event. Process intervals were then defined from the synchronized camera video based on plume location, morphology, ejection direction, and persistence. The photodiode and accelerometer data were subsequently evaluated on this common time axis using the camera-defined process intervals. No quantitative sensor-to-sensor time delay was estimated; these signals were evaluated for response characterization rather than as independent channels for process-state identification.

**Figure 3:**
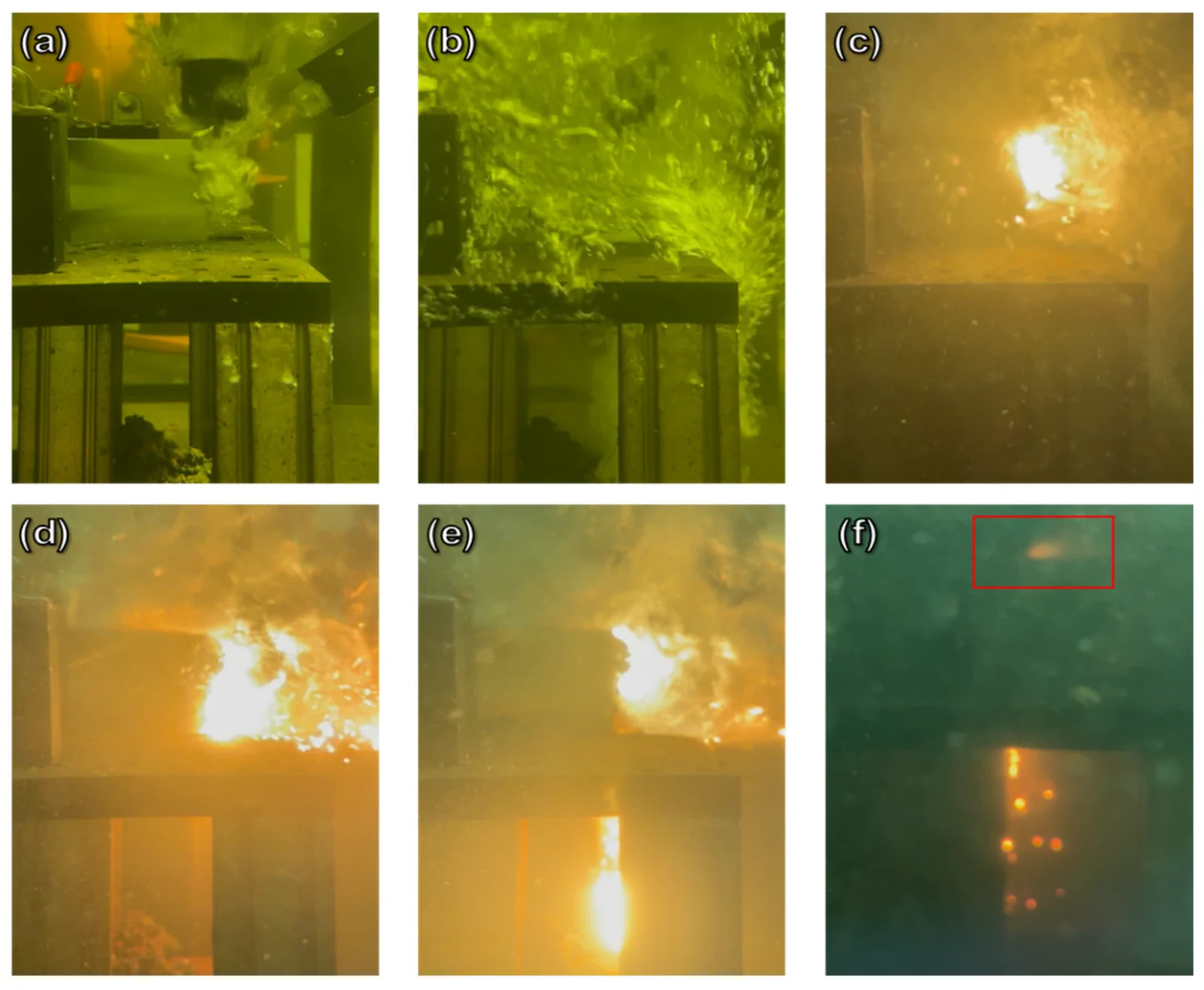
Representative camera images of the cutting process: (**a**) standby condition with N_2_ at 1 bar before laser emission, (**b**) pre-cutting with N_2_ at 5 bar and laser emission before beam–specimen interaction, (**c**) partial penetration, (**d**) transient strong ejection from the lower lateral side immediately before full penetration, (**e**) full penetration, and (**f**) late-stage abnormal behavior. The red box in (**f**) highlights the renewed upper/nozzle-side luminous activity.

Before the synchronized cutting intervals, the camera also captured a standby condition in which N_2_ was discharged at 1 bar before laser emission (Figure 3a). This image is included only as a pre-process reference and was not used as a stage-wise statistical interval. Pre-cutting was defined as the interval in which the N_2_ pressure was increased to 5 bar and the laser was emitting, but the beam had not yet reached the specimen; no cutting-induced luminous plume was present (Figure 3b). Partial penetration was defined as the interval from cutting start, identified by the onset of sustained laser–material interaction and plume formation (Figure 3c), until the onset of full penetration. A short, intense ejection from the lower lateral side of the specimen was observed immediately before full penetration (Figure 3d); this was treated as a transient precursor event within the late partial penetration interval rather than as a separate process state. Full penetration was identified when a through-thickness discharge path formed and sustained downward ejection appeared below the specimen (Figure 3e).

For stage-wise statistics, full penetration was represented by a 1 s interval beginning at this camera-defined onset, while the subsequent 10 s interval was defined as post-penetration. The analysis-window lengths were selected according to the temporal characteristics of the corresponding process states. The 1 s full-penetration interval was used to capture the short-duration, intense downward ejection associated with the initial formation of the through-thickness discharge path while limiting the contribution of the subsequent post-penetration response. The following 10 s interval was used to characterize the immediate post-penetration condition. For Experiments 1 and 3, the cutting process was classified into pre-cutting, partial penetration, full penetration, and post-penetration based on the camera observations described above. In Experiment 2, the same general penetration progression was observed, but an additional late-stage change in plume behavior appeared after full penetration and the subsequent post-penetration period. Therefore, a late-stage abnormal interval was additionally defined for Experiment 2. This interval was identified by a sustained departure from the preceding post-penetration plume behavior, accompanied by renewed upper/nozzle-side plume activity (Figure 3f).

**Figure 4:**
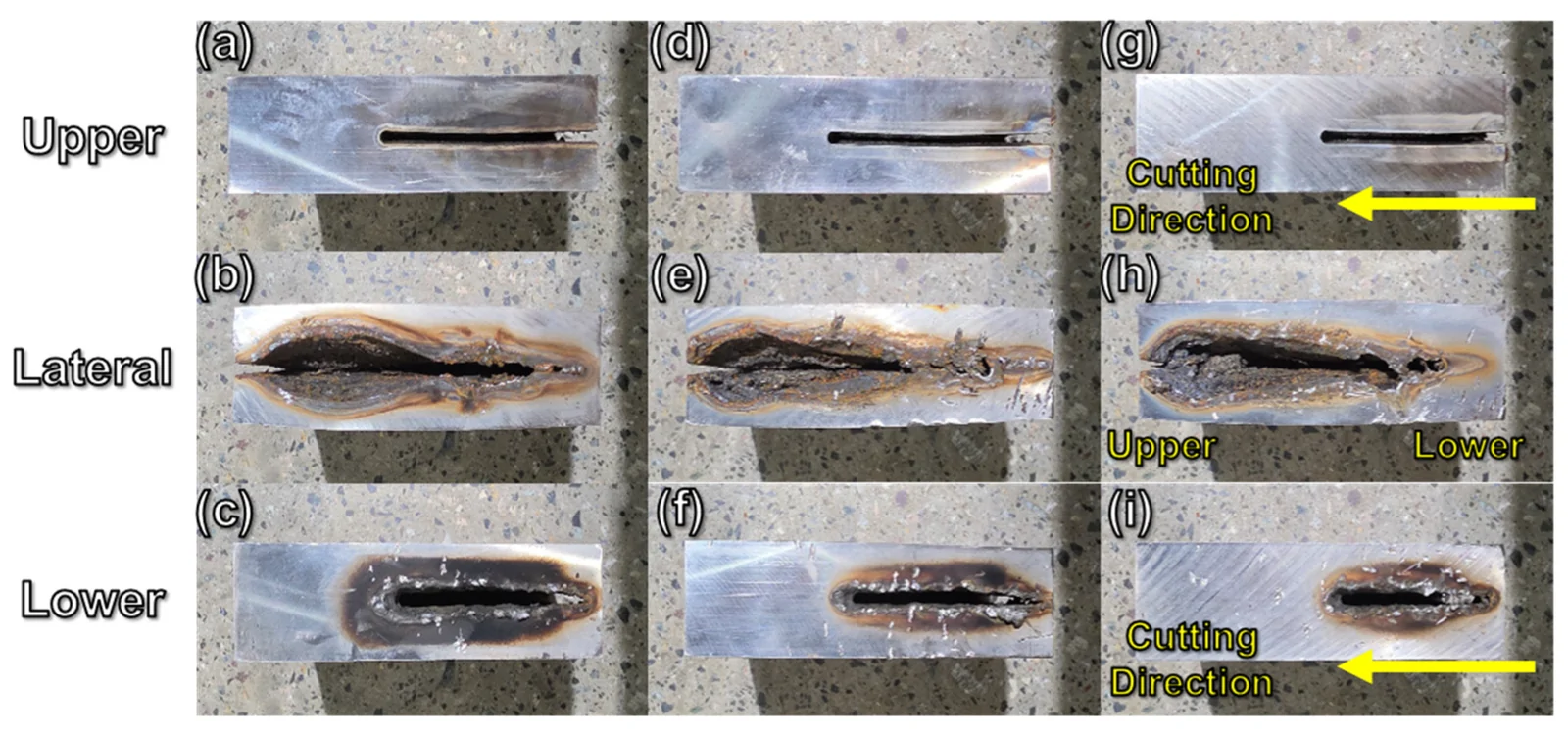
Post-test appearances of the specimens from Experiments 1–3: (**a**–**c**) upper, lateral, and lower surfaces of Experiment 1, respectively; (**d**–**f**) upper, lateral, and lower surfaces of Experiment 2; and (**g**–**i**) upper, lateral, and lower surfaces of Experiment 3.

Post-test inspection confirmed the presence of cut traces on the lower surfaces of all three specimens, providing an independent physical reference that through-thickness penetration had occurred. Representative upper-, side-, and lower-surface views of the specimens are shown in Figure 4. The synchronized analysis intervals for Experiments 1 and 3 are summarized in Table 2.

### 2.3. Multisensor Data Processing and Analysis

Because camera exposure and gain were not fixed during acquisition, the raw image intensity was not treated as an absolute optical intensity. Instead, the images were processed to obtain a relative image response with reduced influence from spatially nonuniform background brightness and to calculate the projected plume area. However, local-background normalization does not completely eliminate the possible influence of automatic exposure and gain adjustments on the derived image metrics. Accordingly, the image-derived quantities were treated as descriptive image metrics for characterizing relative intensity and spatial changes in the observed luminous region rather than as calibrated measurements of optical emission. Each frame was converted to grayscale, and a large-scale Gaussian filter was applied over the full frame to estimate the slowly varying local background B(x,y,t) around each pixel. The background-estimation parameter was set to 200 pixels, corresponding to an effective Gaussian kernel size of 201 × 201 pixels. This kernel size defines the spatial neighborhood used for local-background estimation rather than the Gaussian standard deviation. The same setting was applied to all analyzed videos. The pixel intensity I(x,y,t) was then compared with the locally estimated background at the same position. Because division by a near-zero background can artificially amplify the normalized value in very dark regions, background values below 5 gray levels were clipped to 5 (*B_min_* = 5). The pixel-wise background-normalized value was calculated as

(1) $$r(x,y,t)=\frac{I(x,y,t)}{\max[B(x,y,t),\;B_{\min}]}$$

where x and y denote the spatial pixel coordinates, t denotes time, I(x,y,t) is the grayscale intensity at pixel (x,y) and time t, B(x,y,t) is the corresponding locally estimated background intensity, and *B_min_* is the minimum background value used to prevent division by near-zero values. The resulting r(x,y,t) represents the dimensionless pixel-wise intensity normalized by the local background. A value of 1 in Equation (1) indicates that the pixel intensity is equal to the estimated local background, whereas values greater than 1 indicate pixels brighter than their local background.

Within each ROI, the pixel-wise normalized values were averaged to obtain the ROI to background ratio *R_k_*(*t*):

(2) $$R_{k}(t)\;=\;\frac{1}{N_{k}}\sum_{(x,y)\in {\mathrm{ROI}}_{k}}r(x,y,t)$$

where k denotes the ROI index (upper, lateral, or lower), *ROI_k_* denotes the set of pixels belonging to the k-th ROI, *N_k_* is the number of pixels in that ROI, and *R_k_*(*t*) represents the mean background-normalized response of the k-th ROI at time t.

The projected plume area was segmented from the same background-normalized image. Before thresholding, a 5 × 5 pixel Gaussian smoothing kernel was applied to reduce small-scale intensity fluctuations. A fixed ratio threshold of 1.15 was used as a lower bound to suppress small background-brightness fluctuations; thus, a pixel had to be at least 15% brighter than its estimated local background to be considered a plume candidate. This fixed value was applied uniformly to all analyzed videos. To account for frame-to-frame changes in the brightness distribution, a frame-wise Otsu threshold was also calculated, and the larger of the two thresholds was used for segmentation:

*T*(*t*) = *max*[1.15,*T_Otsu_*(*t*)] (3)

where *T*(*t*) is the final segmentation threshold applied at time t, and *T_Otsu_*(*t*) is the Otsu threshold calculated from the background-normalized image for the corresponding frame.

**Figure 5:**
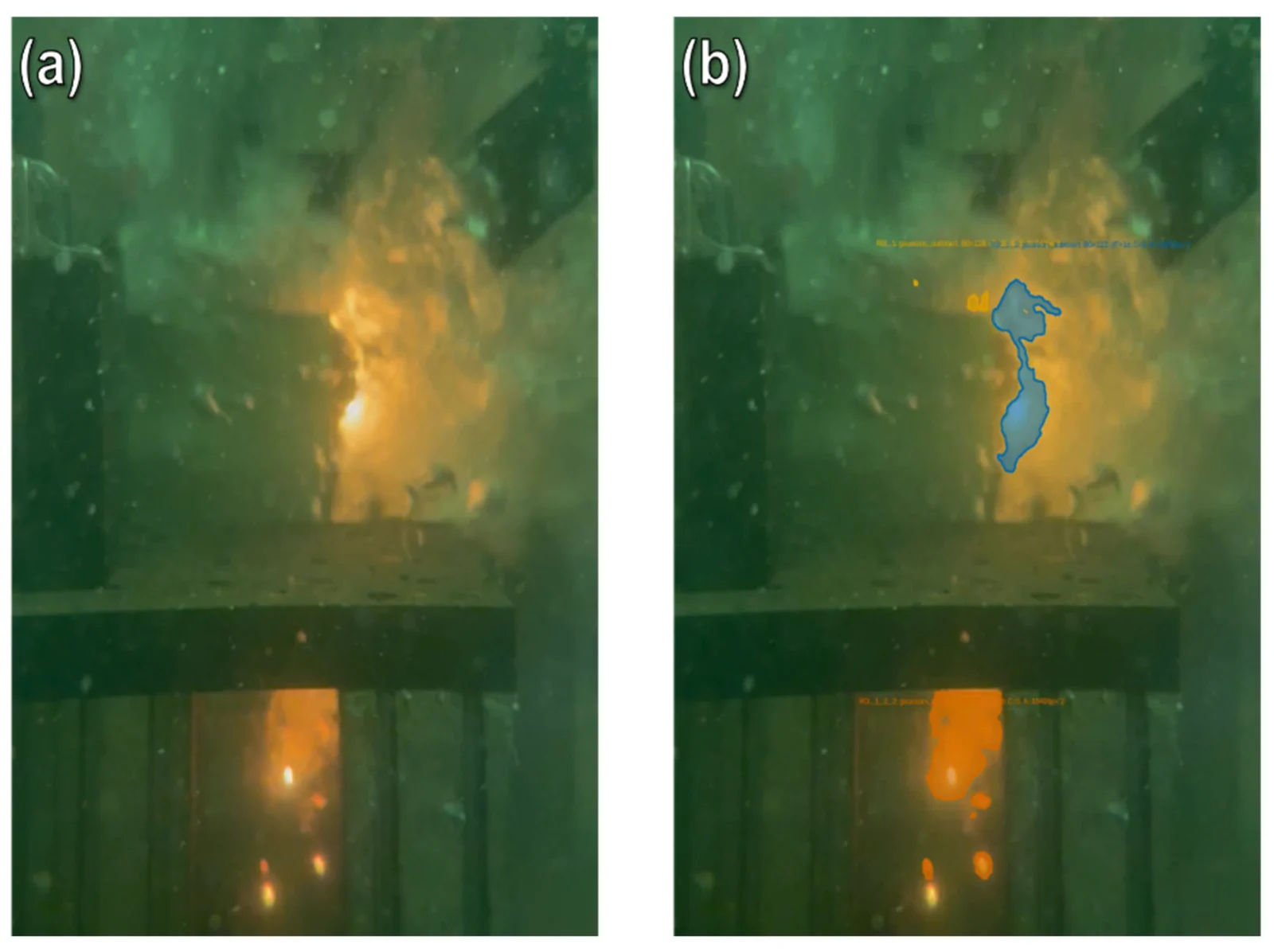
Representative example of plume segmentation during underwater laser cutting: (**a**) original camera image and (**b**) corresponding segmentation overlay obtained using the image-processing procedure described in Section 2.3. The highlighted region in (**b**) represents pixels classified as plume candidates.

After binarization, morphological opening and closing were performed using a 3 × 3 pixel elliptical structuring element to remove isolated noise while limiting excessive modification of the segmented boundaries, and connected components smaller than 20 px^2^ were removed. For the final binary plume mask *M_k_*(*x*,*y*,*t*), the projected plume area was calculated as

(4) $$A_{k}(t)=\sum_{(x,y)\in {\mathrm{ROI}}_{k}}M_{k}(x,y,t)$$

where *M_k_*(*x*,*y*,*t*) is the binary plume mask within the k-th ROI, taking a value of 1 for pixels classified as plume candidates and 0 otherwise. *A_k_*(*t*) is the total number of plume-classified pixels within the k-th ROI at time t. *A_k_*(*t*) is reported in px^2^ and represents the two-dimensional projected plume area in the image rather than a physical three-dimensional plume volume. The processing parameters were selected by visually comparing representative original frames with the corresponding segmentation results to check that the principal luminous regions were retained while nonuniform background variations and isolated noise were suppressed. Individual binary masks were not manually modified or corrected. A representative comparison between the original camera image and the corresponding segmentation overlay is shown in Figure 5.

For visualization, the camera time histories were sequentially filtered using a 9-frame median filter and a 15-frame Savitzky–Golay filter (polynomial order 2). Mean, median, interquartile range (IQR), and 95th percentile (P95) were calculated for each process interval to compare both signal level and distributional characteristics. Mean and median describe the central signal level. The IQR was defined as *Q*_75_ − *Q*_25_ and represents the spread of the central 50% of the distribution, where *Q*_25_ and *Q*_75_ denote the 25th and 75th percentiles, respectively. P95 represents the 95th-percentile response level. For Experiment 2, the mean red-channel value of the segmented plume pixels in the upper ROI was additionally extracted to characterize the late-stage change in the nozzle-side plume.

Because the optical measurement chain was not radiometrically calibrated, the measured photodiode voltage was not interpreted as absolute optical power but was used as a relative broadband optical-response signal for characterizing process-dependent changes. The broadband photodiode signal was baseline-corrected using the median of a stable laser-off interval preceding laser emission:

*P_c_*(*t*) = *P*(*t*) − *median_τ_*_∈_*_β_*[*P*(*τ*)] (5)

where P(t) is the raw photodiode voltage at time t, β denotes the selected stable laser-off baseline interval preceding laser emission, τ is the time variable within β, and *P_c_*(*t*) is the baseline-corrected photodiode signal. The median was used to reduce sensitivity to isolated electrical spikes. Photodiode measurements were available for Experiments 1 and 3, and mean, median, IQR, and P95 were calculated for the pre-cutting, partial penetration, 1 s full penetration, and post-penetration intervals defined in Table 2.

For vibration analysis, only the vertical Z-axis component of the accelerometer signal was used. The Z-axis was selected because it was aligned with the cutting-head and nozzle axis and therefore with the primary direction of the assist-gas jet. Changes in kerf opening and molten-material discharge were expected to alter gas–melt interaction and the resulting mechanical excitation primarily along this direction. The acceleration signal was converted from g to SI units and analyzed at 5000 Hz. The power spectral density (PSD) was calculated over time using a 256-sample Hann window, a 50-sample hop, and a fast Fourier transform (FFT) length of 256. The mean of each analysis window was removed before PSD calculation, and a one-sided, density-scaled PSD was obtained. The density scaling accounted for the Hann-window power in the PSD normalization, and the resulting FFT-bin spacing was 19.53 Hz.

In this study, band-mean PSD was defined as the average of the PSD values within a specified frequency band at each time frame, providing a single representative measure of the vibration level in that band. It was calculated for three nominal frequency bands: 10–75, 75–245, and 245–500 Hz. Because the vibration response under the present experimental configuration was not known a priori, three broad nominal frequency bands were defined after exploratory inspection of the measured spectra and applied consistently to all experiments. The nominal band limits did not necessarily coincide with exact FFT-bin frequencies; therefore, band-mean PSD was calculated using only the discrete FFT bins whose center frequencies fell within each nominal range, without frequency interpolation. A Hann window was used to reduce spectral leakage, with no additional leakage correction. For the b-th frequency band *F_b_*, the band-mean PSD was calculated as

(6) $$P_{b}(t_{m})=\frac{1}{N_{b}}\sum_{f_{i}\in F_{b}}S_{\mathrm{aa}}(f_{i},t_{m})$$

where b denotes the frequency-band index, *F_b_* is the frequency range of the b-th band, *N_b_* is the number of frequency bins contained in *F_b_*, *f_i_* denotes the i-th frequency bin, and *t_m_* denotes the m-th time frame of the PSD analysis. *S_aa_*(*f_i_*,*t_m_*) is the PSD of the Z-axis acceleration signal at frequency bin *f_i_* and time frame *t_m_* and *P_b_*(*t_m_*) represents the band-mean PSD of the b-th frequency band at *t_m_*. Both *S_aa_* and *P_b_* are expressed in ${\left(m/s^{2}\right)}^{2}/\mathrm{Hz}$.

For visualization, the band-mean PSD time histories were smoothed with a 5-sample median filter followed by a 21-sample Savitzky–Golay filter (polynomial order 2). Mean, median, IQR, and P95 were calculated for each camera-defined interval. Experiments 1 and 3 were used to compare band-specific vibration changes from partial penetration to the 1 s full penetration interval, whereas Experiment 2 compared equal 20 s windows before and after the camera-defined late-stage abnormal onset across the three nominal frequency bands. The equal 20 s windows were selected to provide a balanced comparison before and after the camera-defined onset while limiting the inclusion of longer-term process evolution. All reported mean, median, IQR, and P95 values were treated as within-experiment descriptive statistics. Individual time-series samples or video frames were not considered independent experimental replicates; the experiment itself was the unit of replication, and no population-level statistical inference was performed. Because the partial-penetration interval was dynamically evolving and differed in duration between experiments, the corresponding stage-wise statistics were interpreted as descriptive summaries of the entire interval rather than as stationary signal levels.

## 3. Results and Discussion

### 3.1. Spatial Characteristics of the Luminous Response During Cutting

**Figure 6:**
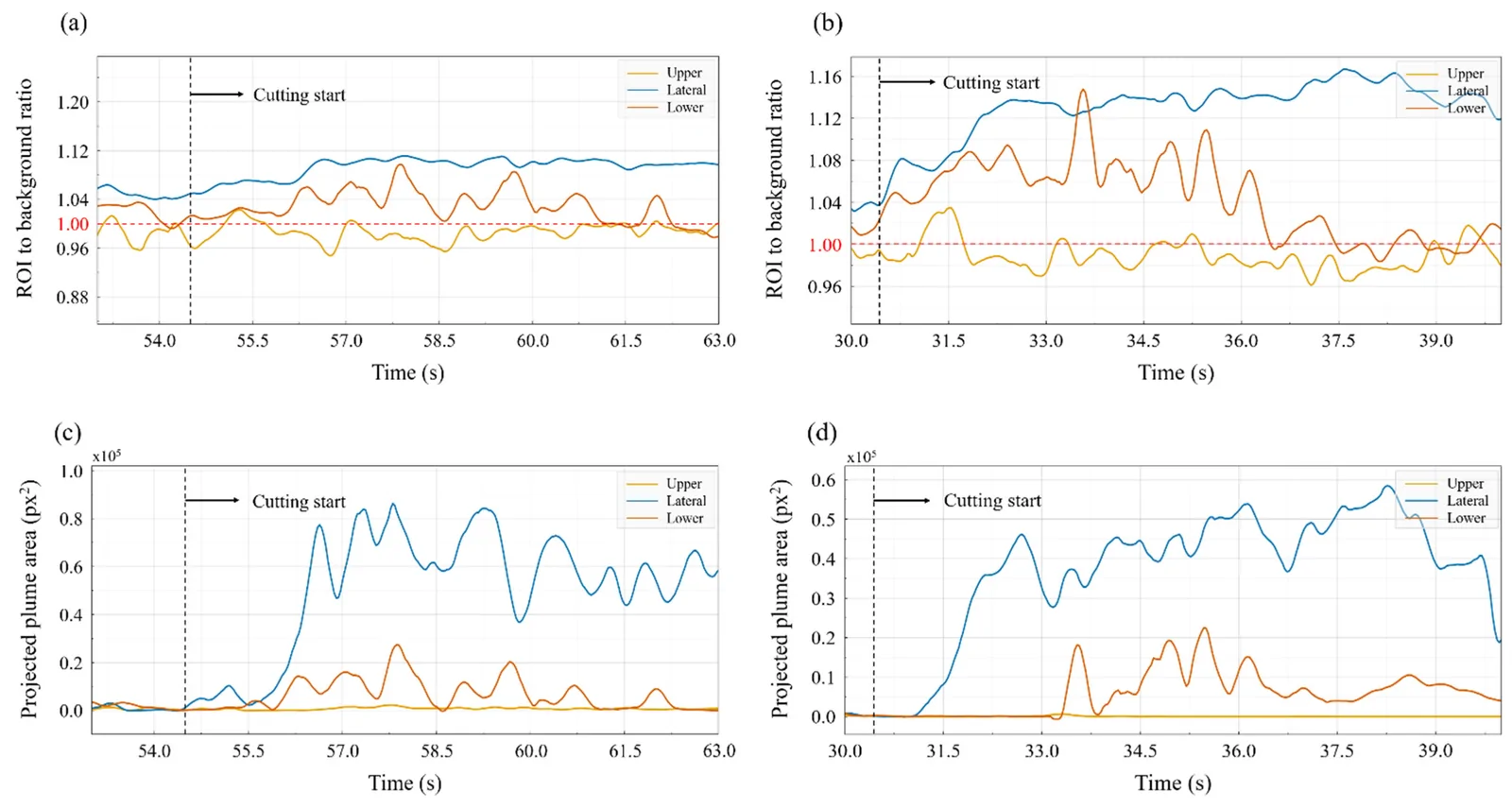
Camera-derived time histories around cutting start. The upper panels show the ROI to background ratio and the lower panels show the projected plume area. (**a**,**c**) Experiment 1 and (**b**,**d**) Experiment 3. The upper, lateral and lower ROIs are shown together. The vertical dashed line denotes the cutting start, identified by the onset of sustained laser–material interaction and plume formation.

**Figure 7:**
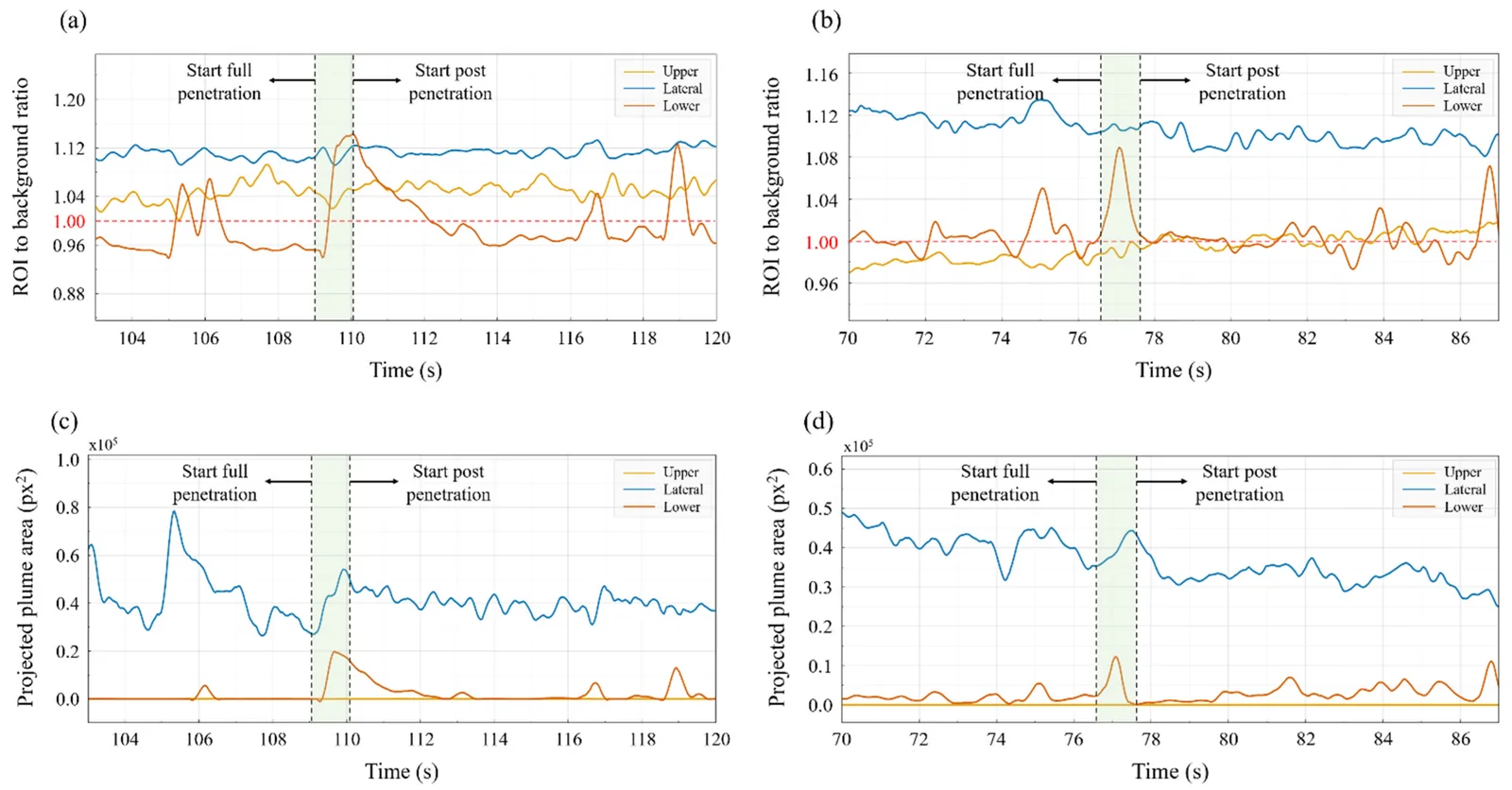
Camera-derived time histories around full penetration. The upper panels show the ROI to background ratio and the lower panels show the projected plume area. (**a**,**c**) Experiment 1 and (**b**,**d**) Experiment 3. The first vertical dashed line denotes the onset of full penetration and the second denotes the onset of post-penetration.

Representative camera frames in Figure 3c–e show the sequence from partial penetration, through the transient precursor ejection immediately before full penetration, to full penetration. Figure 6 and Figure 7 quantify the principal camera-defined transitions in Experiments 1 and 3. The upper panels show the ROI to background ratio for the upper, lateral and lower ROIs, while the lower panels show the projected plume areas extracted from the same ROIs. This arrangement allows the change in mean ROI to background ratio to be compared directly with the change in the spatial extent of the plume on the same time basis. Table 3 summarizes the principal changes observed in Figure 6 and Figure 7 using stage-averaged values.

After cutting start, the most prominent change in both experiments was the rapid formation of a lateral plume. The mean lateral ROI to background ratio increased from 1.054 to 1.099 in Experiment 1 and from 1.060 to 1.129 in Experiment 3, indicating that the lateral region became relatively brighter than its local background after cutting began. The change was substantially more pronounced in the plume area. The mean lateral plume area increased from 447 to 47,440 px^2^ in Experiment 1 and from 2681 to 42,723 px^2^ in Experiment 3. The time histories likewise show that the lateral plume rapidly expanded and remained at a scale of several tens of thousands of pixels, whereas the upper plume remained small and the lower-region response was more intermittent. Thus, partial penetration involved both an increase in the relative lateral image response and a large spatial expansion of the lateral plume, with the latter providing the clearer image signature of this transition.

This lateral plume expansion is interpreted as a consequence of material-removal behavior before full penetration. In fusion cutting, the N_2_ assist gas transfers momentum to the molten layer and contributes to melt removal and kerf formation. As penetration progressed toward full penetration, a continuous discharge path to the lower surface had not yet been fully established; therefore, molten material and hot luminous ejecta were more broadly expelled around the lateral cutting region rather than being discharged directly beneath the specimen, producing the large increase in lateral plume area.

Around full penetration as shown in Figure 7, both experiments showed a distinct precursor ejection from the lower lateral side of the specimen, corresponding to the behavior represented in Figure 3d, followed by a much larger downward discharge at full penetration (Figure 3e). In Experiment 1, a short and intense molten-material ejection was observed at approximately 105 s, and a similar event occurred at approximately 75 s in Experiment 3. These events subsided temporarily, after which substantially larger molten-material and luminous-plume discharge developed beneath the specimen during the camera-defined full penetration interval (Figure 7). The stage-averaged lower ROI to background ratio increased from 0.989 to 1.065 in Experiment 1 and from 1.012 to 1.045 in Experiment 3. Simultaneously, the lower projected plume area increased from 460 to 5729 px^2^ and from 2982 to 5667 px^2^, respectively. Thus, full penetration was accompanied by increases in both the spatial extent and the mean relative image response of the lower-region plume.

This temporal sequence can be interpreted as a transient process in which localized melt ejection occurs before a continuous through-thickness discharge path becomes established. The short lower-lateral ejection preceding full penetration may represent an initial local release of molten material, whereas opening of the through-thickness kerf allows the N_2_ assist gas and molten or vaporized material to be discharged directly beneath the specimen [8]. The pronounced lower-side burst at full penetration is therefore interpreted as a short clearing event in which molten material and hot gas formed before penetration are rapidly expelled through the newly opened path.

Immediately after the large downward discharge at full penetration, the lower-region luminous plume decreased markedly for a short period and then gradually reappeared. The initial large burst can be interpreted as a clearing event caused by the rapid removal of molten material present when the through-thickness path first opens. Immediately afterward, the amount of material available for discharge is temporarily reduced while the gas flow and molten layer within the kerf transition toward the post-penetration state. As cutting continues, newly generated molten material is removed through the established downward discharge path, leading to the gradual reappearance of lower-side ejection. At the same time, the mean lateral plume area decreased from 47,440 px^2^ during partial penetration to 34,115 px^2^ during post-penetration in Experiment 1 (−28.1%) and from 42,723 to 32,433 px^2^ in Experiment 3 (−24.1%). In contrast, the lateral ROI to background ratio changed in opposite directions, increasing slightly from 1.099 to 1.117 in Experiment 1 and decreasing from 1.129 to 1.097 in Experiment 3. The transition to post-penetration was therefore expressed more consistently by contraction of the lateral plume than by a change in its mean relative image response.

Overall, the camera observations and time histories showed a sequential progression from a broad and sustained lateral plume during partial penetration to a short, intense lower-lateral molten-material ejection immediately before full penetration, followed by a large downward discharge when full penetration was reached. The lower-region luminous plume then decreased temporarily and gradually re-formed, while the lateral plume contracted during post-penetration. This temporal progression indicates that the transient precursor ejection, formation of the through-thickness discharge path, and subsequent transition to post-penetration discharge behavior were reflected in the camera observations and quantitative image metrics.

### 3.2. Photodiode Response to Full Penetration

**Figure 8:**
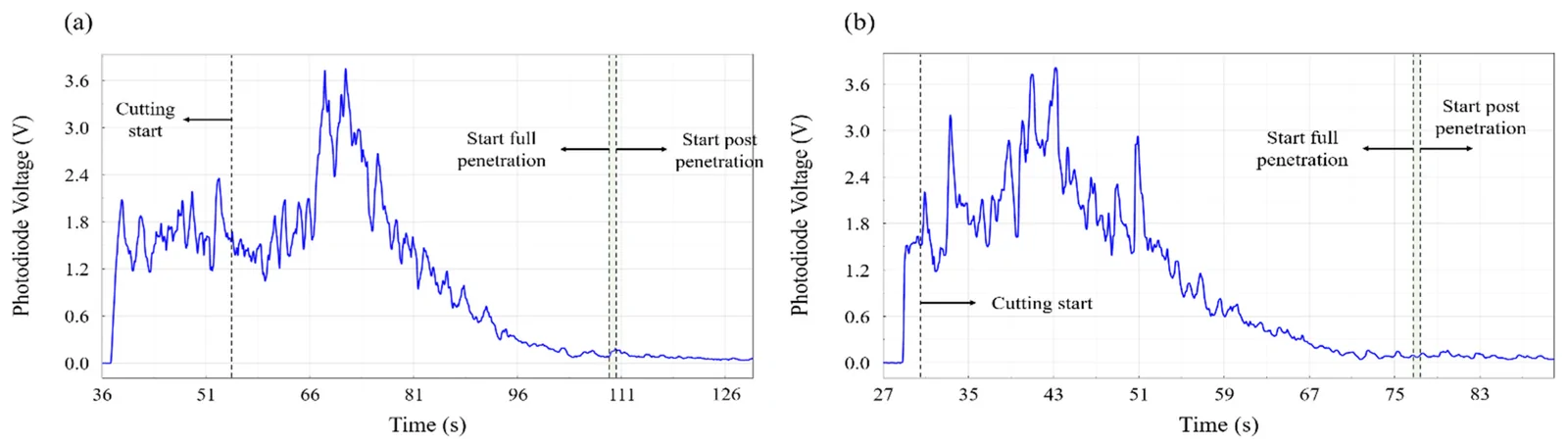
Baseline-corrected photodiode time histories with camera-defined process boundaries. (**a**) Experiment 1. (**b**) Experiment 3.

Photodiode measurements were available for Experiments 1 and 3. Figure 8 presents the complete photodiode time histories together with the camera-defined process intervals. In the pre-cutting interval preceding cutting start, a relatively high photodiode response was observed in both experiments. Although a cutting-induced plume had not yet formed, laser emission had already begun while the N_2_ assist gas maintained a local dry zone. The measured signal may therefore have included optical components associated with laser interaction with residual water or vapor around the local dry zone, scattering at the gas–water interface, or reflections from surrounding surfaces. After cutting start, during the partial-penetration interval, the signal fluctuated over a broad range and its overall level progressively decreased as the process approached full penetration. By the time full penetration was reached, the signal had converged to a low and narrow range, and this low-response regime persisted during post-penetration.

Table 4 summarizes the stage-wise photodiode statistics and shows the changes in signal level and distribution as the process progressed from partial to full penetration. During partial penetration, the IQR values were 1.477 and 1.681 and the P95 values were 3.255 and 2.915 in Experiments 1 and 3, respectively, indicating broad response distributions. During full penetration, the mean decreased from 1.233 to 0.138 in Experiment 1 and from 1.282 to 0.090 in Experiment 3. The IQR decreased from 1.477 to 0.071 and from 1.681 to 0.023, corresponding to reductions of 95.2% and 98.6%, respectively. The P95 likewise decreased from 3.255 to 0.313 and from 2.915 to 0.123, corresponding to reductions of 90.4% and 95.8%. Thus, by the full penetration interval, the central signal level had decreased while the response distribution had also narrowed markedly.

The temporal decrease can be interpreted together with the photodiode viewing geometry and the camera-observed spatial evolution of the plume. During partial penetration, a broad and strongly fluctuating luminous plume and hot ejecta were observed around the lateral and upper regions while the photodiode signal progressively decreased as cutting proceeded. Near the end of partial penetration, the transient lower-lateral precursor ejection represented in Figure 3d was observed, followed by the larger downward discharge at camera-confirmed full penetration (Figure 3e). Once full penetration was established, the downward discharge path remained present. This spatial redistribution may have reduced the luminous component collected along the photodiode viewing direction. However, the broadband photodiode response may also have been influenced by changes in process emission, underwater optical scattering, viewing geometry, and local dry-zone behavior. Importantly, the photodiode decline began before the camera-confirmed full penetration interval, indicating that the signal may reflect the optical evolution toward penetration rather than only the instant at which penetration is visually confirmed.

The photodiode response remained low during post-penetration. The mean changed from 0.138 during full penetration to 0.095 during post-penetration in Experiment 1 and from 0.090 to 0.085 in Experiment 3. The post-penetration IQR remained low at 0.041 and 0.029, respectively, and the corresponding P95 values were 0.172 and 0.136. Thus, the low signal level and narrow distribution observed near full penetration were not confined to the 1 s transition interval but persisted after penetration.

In both experiments, partial penetration was characterized by a broad response distribution together with a progressive downward trend as cutting proceeded. By the time full penetration was reached, the signal had transitioned to a low and narrow distribution, and this low-response regime persisted during post-penetration. The temporal signal decrease during partial penetration and the contraction of the response distribution near full penetration were therefore the main photodiode features observed in the present data.

### 3.3. Accelerometer Response During Penetration and Late-Stage Abnormal Behavior

**Figure 9:**
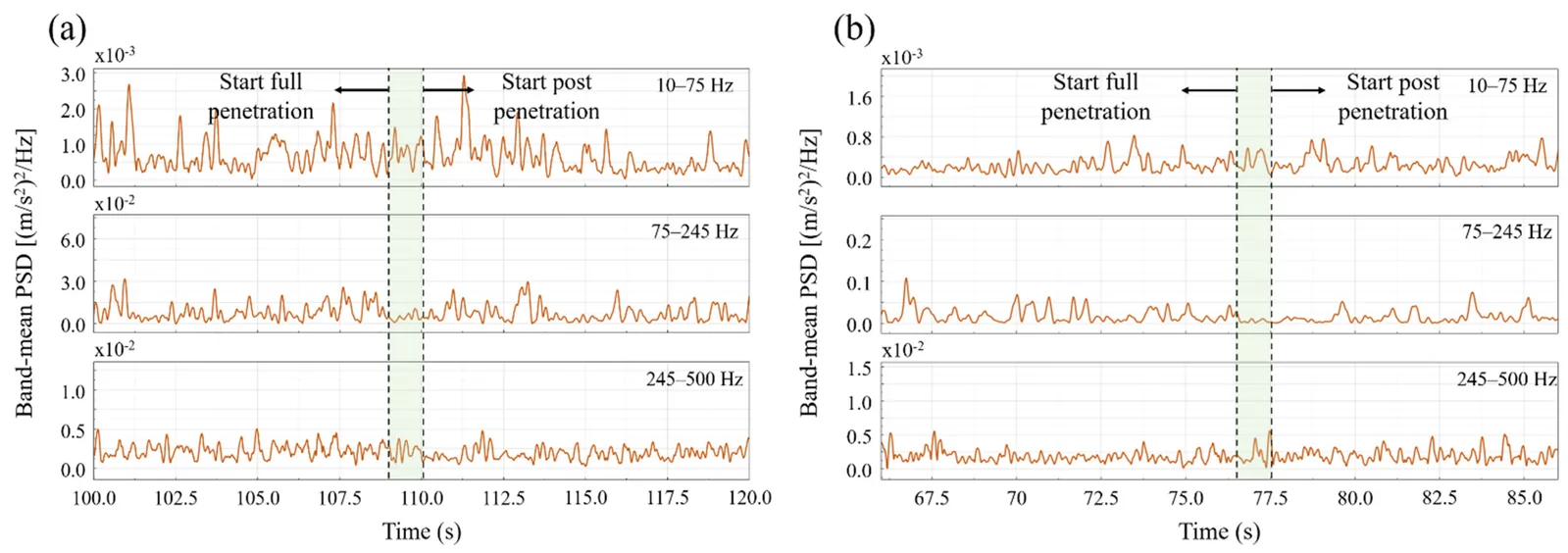
Z-axis band-mean PSD time histories across the full-penetration transition. (**a**) Experiment 1. (**b**) Experiment 3. The vertical dashed lines indicate the start of full penetration and the start of post-penetration, and the interval between them represents the 1 s full-penetration interval.

The Z-axis band-mean PSD time histories around full penetration in Experiments 1 and 3 are shown in Figure 9. Stage-wise comparison revealed some common band-specific changes from partial to full penetration in the two experiments. In particular, the mean PSD in the 75–245 Hz band decreased by 44.4% and 23.5% from partial to full penetration in Experiments 1 and 3, respectively, while the median decreased by 33.9% and 34.9%. The mean PSD in the 10–75 Hz band increased by 25.0% and 35.0%, respectively.

However, although the stage-wise statistics showed common directions of change in the 75–245 Hz and 10–75 Hz bands in both experiments, these changes were not expressed as distinct step-like transitions in the time histories. The absolute PSD levels and effect magnitudes differed between experiments, and the IQR and P95 did not exhibit the same changes in both runs. These results indicate that the accelerometer contained band-specific responses associated with full penetration, but with greater run-to-run variability than the photodiode signal. The accelerometer response around full penetration was therefore treated as a secondary mechanical observation rather than as a primary indicator of the penetration transition.

**Figure 10:**
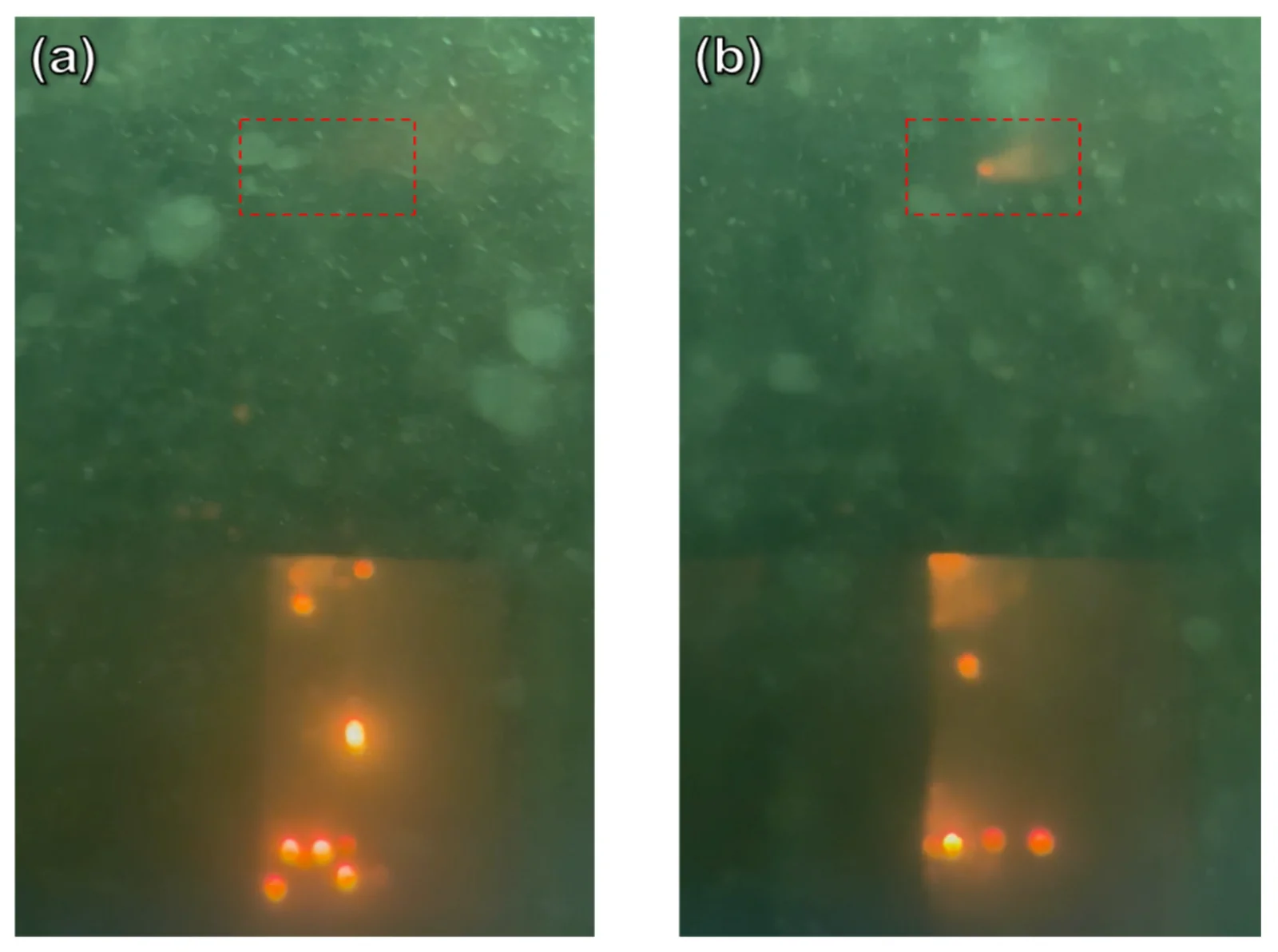
Representative camera images before and during late-stage abnormal behavior in Experiment 2. (**a**) Post-penetration condition preceding the abnormal onset. (**b**) Late-stage abnormal condition showing renewed upper/nozzle-side luminous activity. The red dashed boxes indicate the upper/nozzle-side region used for visual comparison.

**Figure 11:**
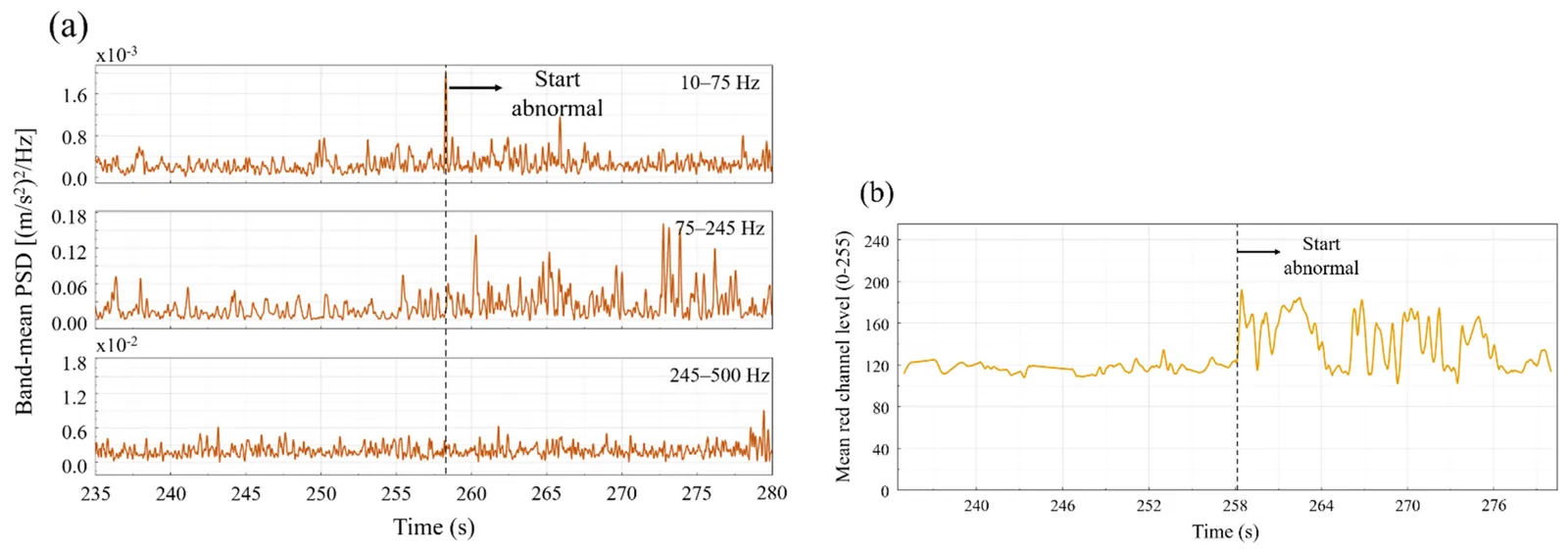
Synchronized responses during late-stage abnormal behavior in Experiment 2. (**a**) Z-axis band-mean PSD in the 10–75, 75–245, and 245–500 Hz ranges. (**b**) Mean red-channel response of the plume region in the Upper ROI. The vertical dashed line indicates the camera-defined onset of late-stage abnormal behavior at approximately 258.3 s.

By comparison, Experiment 2 exhibited a substantially larger change in the 75–245 Hz band during late-stage abnormal behavior. Representative camera images before and during this behavior are compared in Figure 10. Compared with the preceding post-penetration condition (Figure 10a), the late-stage abnormal condition showed renewed upper/nozzle-side luminous activity (Figure 10b). Using the camera-defined onset at approximately 258.3 s, the preceding 20 s post-penetration interval (238.3–258.3 s) was compared with the subsequent 20 s late-stage abnormal interval (258.3–278.3 s). Based on the actual cutting start and the programmed feed profile, the cutting head was located approximately 39.4 mm from the starting edge at this onset, within the 30 mm/min cutting-speed region. Post-test measurements showed an upper-surface cut trace of approximately 50.0 mm and a lower-surface cut termination at approximately 46.0 mm, indicating that local through-thickness geometric differences were present in the later part of the cutting path. Because the photodiode record was unavailable in Experiment 2, the synchronized comparison was limited to the camera and accelerometer signals. Figure 11 presents the Z-axis band-mean PSD and the upper-ROI red-channel response on the same time basis, with the camera-defined abnormal onset marked in both panels.

The most pronounced change occurred in the 75–245 Hz band. As shown in Figure 11a, more frequent high-PSD peaks appeared after 258.3 s in this band, whereas comparable sustained changes were not evident in the other two bands. The mean PSD increased from 0.01372 to 0.03139 (m/s^2^)^2^/Hz, corresponding to an increase of 128.8%. The median, IQR, and P95 increased by 125.1%, 106.7%, and 120.8%, respectively. In contrast, the 10–75 Hz mean increased by only 24.9% and the 245–500 Hz mean by 1.8%; the IQR and P95 in the latter band slightly decreased. The response was therefore frequency-selective rather than a uniform broadband rise in vibration.

The camera response in Figure 11b changed at the same camera-defined onset. The mean red-channel value of the segmented plume pixels in the Upper ROI increased from 117.6 during the preceding post-penetration interval to 145.4 during the late-stage abnormal interval, corresponding to an increase of 23.6%. The IQR increased from 6.01 to 44.07, corresponding to an increase of 633.3%. The increase in IQR represents a widening of the within-window response distribution and is not, by itself, interpreted as temporal instability. Together with the visual comparison in Figure 10, these synchronized changes indicate that the renewed upper/nozzle-side plume activity coincided with increased mechanical excitation concentrated primarily in the 75–245 Hz band. The principal camera and accelerometer changes associated with this late-stage abnormal behavior are summarized in Table 5.

The renewed upper/nozzle-side plume activity may reflect a temporary disturbance in the established downward molten-material discharge path. Sparkes et al. reported that insufficient melt removal during inert-gas laser cutting of 304 stainless steel can result in upward melt ejection from the top surface and recast or dross formation in the lower region. Their CFD analysis further showed that assist-gas flow characteristics within the kerf play an important role in melt-ejection behavior [15]. Based on these findings, the late-stage upper/nozzle-side luminous activity observed in Experiment 2 may be associated with restricted or disturbed downward melt ejection. Such a change in gas–melt interaction and material discharge could also increase the mechanical excitation transmitted to the cutting head, which is consistent with the pronounced increase observed in the 75–245 Hz band. Therefore, the synchronized camera and accelerometer responses in Experiment 2 suggest that the late-stage abnormal behavior may be associated with disturbed molten-material discharge rather than a general increase in vibration across all frequency ranges.

## 4. Conclusions

This study synchronized camera, photodiode, and accelerometer signals during underwater laser cutting of 100 mm thick STS 304 to analyze process-state-dependent optical and mechanical changes and to evaluate sensor features associated with full penetration and late-stage abnormal behavior. The main conclusions are summarized as follows.

1. Camera analysis showed a broad and sustained lateral plume during partial penetration, followed by a short, intense lower-lateral ejection immediately before full penetration. At full penetration, both the ROI to background ratio in the lower ROI and plume area increased markedly relative to the preceding partial penetration state, together with a pronounced downward discharge. After penetration, the lateral plume area decreased in both experiments. These similar spatial changes observed in both experiments indicate that the distribution of luminous and ejection regions changes as penetration progresses through the thickness and that image-based features of the process transitions can be quantitatively described.
2. In both photodiode experiments, the signal exhibited a broad response distribution during partial penetration and progressively decreased as full penetration was approached. By the full penetration interval, the response had transitioned to a low and narrow distribution. The mean signal decreased by 88.8% and 93.0% from partial to full penetration in Experiments 1 and 3, respectively, while the IQR decreased by 95.2% and 98.6%. The low-response, narrow-distribution regime persisted during post-penetration. These results indicate that the photodiode was more informative for tracking the temporal signal decrease and distribution change associated with progression toward full penetration than for distinguishing states by their absolute stage-average level.
3. Some common directional changes in the band-dependent accelerometer responses were observed around full penetration in Experiments 1 and 3, although their magnitudes varied between experiments. The camera-defined late-stage abnormal behavior was characterized by renewed upper/nozzle-side plume activity, and the 75–245 Hz mean PSD increased by 128.8% relative to post-penetration, while the median, IQR, and P95 increased by 125.1%, 106.7%, and 120.8%, respectively. In contrast, the 10–75 Hz mean PSD increased by only 24.9%, and the 245–500 Hz mean PSD changed by only about 1.8%. Thus, in Experiment 2, the observed mechanical response during late-stage abnormal behavior was not a uniform broadband increase but showed its largest change in the 75–245 Hz band.

Overall, the three sensors did not simply reproduce the same process change; rather, they exhibited sensitivity to different aspects of the cutting process. These differences suggest a basis for assigning distinct roles to individual sensors in future multisensor monitoring. In underwater dismantling environments where continuous visual observation may be limited, non-imaging sensors such as photodiodes and accelerometers may provide complementary information on process-state changes. However, the present results characterize sensor-response tendencies and do not demonstrate reliable or automatic process-state detection. These findings may provide an experimental basis for the future development and validation of an underwater laser-cutting monitoring system. Given the limited number of observations, future work should expand the number of repeated trials and the range of material thicknesses and process conditions to verify the reproducibility of the identified sensor features and evaluate the generalizability of multisensor state-monitoring approaches.

## Figures and Tables

**Table 1:** Principal process conditions used in the underwater laser-cutting experiments.

Category	Parameter	Setting
Workpiece	Material	STS 304 stainless steel
	Thickness	100 mm
Laser	Source	Fiber laser
	Rated output	30 kW
	Cutting power	22 kW
Cutting motion	Cutting speed, path position 0–30 mm	12 mm min^−1^
	Cutting speed, path position >30 mm	30 mm min^−1^
Optical setup	Focal position	−40 mm (40 mm below upper surface)
Assist gas	Gas	N_2_
	Pressure	5 bar
Cutting head	Nozzle-to-specimen standoff	5 mm
Environment	Water depth	1 m

**Table 2:** Camera-defined process states and synchronized analysis intervals for Experiments 1 and 3.

Process State/Event	Video-Based Definition	Experiment 1	Experiment 3
Pre-cutting	N_2_ at 5 bar and laser on; beam has not yet reached the specimen; no cutting-induced luminous plume.	37.64–54.47 s	28.70–30.44 s
Partial penetration	Sustained plume after laser–material interaction; interval before full penetration, including the precursor ejection in Figure 3d.	54.47–109.07 s	30.44–76.61 s
Full penetration	Camera-confirmed onset of sustained through-thickness downward ejection; 1 s interval used for statistics.	109.07–110.07 s	76.61–77.61 s
Post-penetration	Cutting interval immediately after the 1 s full penetration interval.	110.07–120.07 s	77.61–87.61 s

**Table 3:** Principal camera-derived changes during penetration progression.

Metric	Experiment 1	Experiment 3
Lateral ROI to background ratio, Pre → Partial	1.054 → 1.099	1.060 → 1.129
Lateral plume area, Pre → Partial (px^2^)	447 → 47,440	2681 → 42,723
Lower ROI to background ratio, Partial → Full	0.989 → 1.065	1.012 → 1.045
Lower plume area, Partial → Full (px^2^)	460 → 5729 (+1145.4%)	2982 → 5667 (+90.0%)
Lateral plume area, Partial → Post (px^2^)	47,440 → 34,115 (−28.1%)	42,723 → 32,433 (−24.1%)

**Table 4:** Stage-wise photodiode statistics. IQR denotes the Q75–Q25 range, and P95 denotes the 95th percentile. All values are given in V.

Experiment	Interval	Mean	Median	IQR	P95
Exp. 1	Pre-cutting	1.604	1.482	0.633	2.609
	Partial penetration	1.233	1.089	1.477	3.255
	Full penetration	0.138	0.111	0.071	0.313
	Post-penetration	0.095	0.084	0.041	0.172
Exp. 3	Pre-cutting	1.308	1.521	0.148	1.635
	Partial penetration	1.282	1.304	1.681	2.915
	Full penetration	0.090	0.088	0.023	0.123
	Post-penetration	0.085	0.081	0.029	0.136

**Table 5:** Changes associated with late-stage abnormal behavior in Experiment 2. Camera red-channel values are reported as 8-bit intensity levels (0–255), and PSD values are reported in (m/s^2^)^2^/Hz.

Signal/Metric	Post-Penetration	Late-Stage Abnormal	Change (%)
Upper ROI red-channel mean	117.59	145.39	+23.6%
Upper ROI red-channel IQR	6.01	44.07	+633.3%
10–75 Hz PSD mean	2.33 × 10^−4^	2.91 × 10^−4^	+24.9%
75–245 Hz PSD mean	1.372 × 10^−2^	3.139 × 10^−2^	+128.8%
75–245 Hz PSD median	1.033 × 10^−2^	2.325 × 10^−2^	+125.1%
75–245 Hz PSD IQR	1.447 × 10^−2^	2.991 × 10^−2^	+106.7%
75–245 Hz PSD P95	3.784 × 10^−2^	8.354 × 10^−2^	+120.8%
245–500 Hz PSD mean	2.025 × 10^−3^	2.061 × 10^−3^	+1.8%

## Data Availability

The raw/processed data required to reproduce these findings cannot be shared at this time as the data also form part of an ongoing study.
